# Diffusion Tensor Imaging Studies on Recovery of Injured Optic Radiation: A Minireview

**DOI:** 10.1155/2020/8881224

**Published:** 2020-06-09

**Authors:** Eun Bi Choi, Sung Ho Jang

**Affiliations:** Department of Physical Medicine and Rehabilitation, College of Medicine, Yeungnam University, Daemyungdong, Namku, Daegu 705-717, Republic of Korea

## Abstract

The optic radiation (OR) is a visual neural fiber pathway for the transfer of visual information from the lateral geniculate body of the thalamus to the primary visual cortex. To demonstrate the recovery of an OR injury, quantification and visualization of changes to the injured OR are necessary. Diffusion tensor imaging (DTI) allows determination of the state of an OR by assessing the obtained DTI parameters. In particular, diffusion tensor tractography (DTT), which is derived from DTI data, allows three-dimensional visualization of the OR. Thus, recovery of an injured OR can be demonstrated by examining changes in DTI parameters and/or configuration on follow-up DTI scans or via DTT of the injured OR. Herein, we review nine DTI-based studies that demonstrated recovery of OR injuries. The results reported in these studies suggest that an OR injury has a potential for recovery. Moreover, the results of these studies can form a basis for elucidating the recovery mechanisms of injured OR. These studies have suggested two recovery mechanisms for OR injury: recovery via the original OR pathway or via the transcallosal fibers of the corpus callosum. However, only nine studies on this topic have been conducted to date and six of those nine studies were case reports. Therefore, further studies involving larger numbers of subjects and reporting precise evaluations of changes in OR injury during recovery are warranted.

## 1. Introduction

The optic radiation (OR), which is also referred to as the geniculocalcarine tract or the geniculostriate pathway, is a system of vision-related neural fiber pathways from the lateral geniculate body of the thalamus to the primary visual cortex that passes around the lateral aspect of the posterior portion of the lateral ventricle [1]. The OR transfers visual information from the lateral geniculate body to the primary visual cortex, and visual field defects are a typical clinical feature of an injured OR [1].

Knowledge of the recovery process of an injured neural tract in patients with brain injury is clinically important because such knowledge can provide a scientific basis for describing the recovery mechanisms of such tracts; such knowledge is essential for the development of scientifically based effective neurorehabilitation strategies. To demonstrate recovery of an injured OR, quantification and visualization of changes in the injured OR are necessary. Unfortunately, the OR is not clearly distinguishable from adjacent neural structures on conventional brain magnetic resonance imaging (MRI); thus, precise determination of recovery of an injured OR is limited when using conventional brain MRI [2, 3].

Diffusion tensor imaging (DTI), which is a recently developed MRI-based technique, allows determination of the state of an OR based on the obtained DTI parameters; moreover, diffusion tensor tractography (DTT), which is derived from DTI data, can be used to create three-dimensional images suitable for visualization of the state of the OR [4–11]. As a result, recovery of an injured OR can be demonstrated by assessing DTI parameter changes on follow-up DTIs or by examining structure configuration or DTT parameter changes on follow-up DTTs of an injured OR. Since the introduction of DTI, many studies have demonstrated OR injuries in various brain pathologies [12–15]. However, only a few studies using DTI or DTT have demonstrated recovery of an injured OR [16–24].

**Figure 1:**
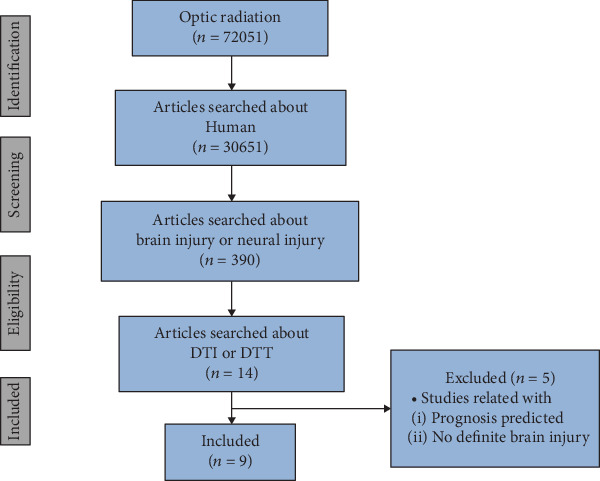
Flow diagram of optic radiation study selection (DTI: diffusion tensor imaging; DTT: diffusion tensor tractography).

In this minireview, DTI studies that have demonstrated recovery of OR injuries are reviewed [16–24]. Relevant studies in the period from 1992 to 2019 were identified by searching the following electronic databases: PubMed, Google Scholar, and MEDLINE. The following keywords and abbreviations were used to search the databases: DTI, DTT, OR, visual recovery, brain plasticity, neural brain injury, stroke, and cerebral infarct. This review was limited to studies of humans. We selected relevant studies according to the flow diagram shown in Figure 1. In total, nine studies were selected and reviewed (Table 1) [16–24].

## 2. Diffusion Tensor Imaging of the Optic Radiation

DTI allows evaluation of white matter tracts by virtue of its ability to visualize differences in water diffusion characteristics [4, 5, 7]. In normal white matter, water molecules move relatively freely in a direction parallel to the nerve fiber tract; however, their cross-tract movements are restricted, causing diffusion anisotropy of the white matter [4, 5, 7]. Among the various DTI parameters, fractional anisotropy (FA), apparent diffusion coefficient (ADC), and fiber voxels (FV) have been used in studies demonstrating the recovery of OR injuries in patients with brain injury [4, 6, 7, 25, 26]. The FA parameter, which indicates the degree of directionality of water diffusion, is used to assess the degree of directionality and integrity of white matter microstructures such as axons, myelin, and microtubules [4, 7]. The ADC is an indicator of the magnitude of water diffusion, and it may be increased in some pathologies, particularly in vasogenic edema and accumulation of cellular debris from axonal damage [4, 7]. The FV which is a similar terminology with the tract volume is an indicator of the number of image voxels that are included in a neural tract [6, 25]. Among those parameters, increments in FA or FV values and/or a decrement in the ADC value on follow-up DTI scans of an injured OR suggest that the injured OR is undergoing recovery.

**Figure 2:**
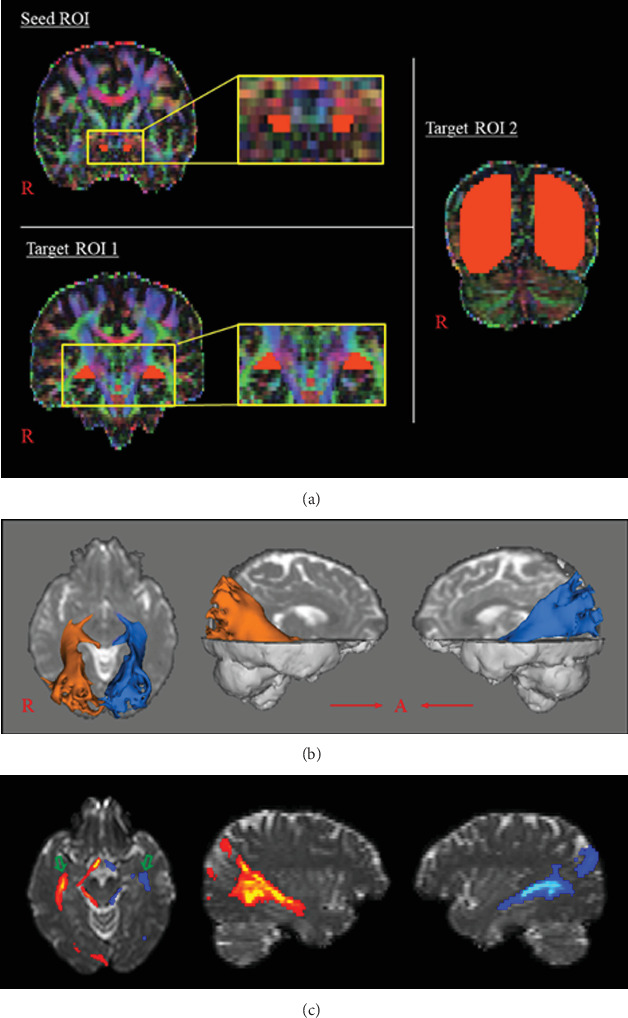
Reconstruction of the optic radiation (OR) using a probabilistic algorithm (the Oxford Centre for Functional Magnetic Resonance Imaging of Brain Software Library; http://www.fmrib.ox.ac.uk/fsl). (a) Regions of interest (ROIs): seed ROI, the optic tract posterior to the optic chiasm in the coronal plane; target ROI 1, the lateral geniculate body in the coronal plane; target ROI 2, the bundle of the OR at the posterior one-third portion between the lateral geniculate body and the primary visual cortex. (b) Results of diffusion tensor tractography of the OR. (c) Axial and sagittal slices of probabilistic tractography show reconstruction of the OR with Meyer's loop (green arrows).

Three methods employed to determine the presence of recovery of an injured OR have been described in previous studies: (1) detection of visual changes on the color maps of follow-up DTIs; (2) detection of changes in the FA value measured in a specific region of interest (ROI) of an injured OR; and (3) detection of changes in the FN value and in three-dimensionally reconstructed configurations on follow-up DTTs [16–24] (Figure 2). However, the method used to detect changes to an injured OR visually on DTI color maps can be subjective, and the ROI-based method can yield false results due to interanalyzer variability in the assigning of ROI location [27]. By contrast, DTT has a distinct advantage as it allows the evaluation of the entire OR in terms of both DTI parameters and tract configuration. DTT algorithms can be classified into two types: deterministic and probabilistic [28]. A deterministic algorithm is reported to be less accurate than a probabilistic algorithm for reconstruction of Meyer's loop, which passes anteroinferiorly over the temporal horn of the lateral ventricle after originating from the lateral geniculate body [28–30]. Moreover, a deterministic algorithm may underestimate fiber tracts in regions of fiber complexity and fiber crossing, which can prevent full reflection of the underlying fiber architecture, whereas a probabilistic algorithm can produce lower specificity resulting in false positives [28, 31–33].

## 3. Previous Diffusion Tensor Imaging Studies on Recovery of Injured Optic Radiations

The first OR injury-related study presented after the introduction of DTI was reported by Seghier et al. in 2005 [16]. The authors reported on a patient who showed OR injury recovery based on their functional MRI (fMRI) and DTI (1.5T INTERA system (Philips Medical Systems, Best, Netherlands) using spin-echo echo-planar imaging (SE-EPI) sequence with 6 noncollinear gradient directions plus one nondiffusion-weighted B0 image) assessments [16]. The infant in that study was reported to develop apneic episodes and focal seizures on the third day after birth at the 37th week of gestation, and a large left-side temporo-parieto-occipital infarct was observed on brain MRI on day four after birth [34]. Three months after birth, fMRI revealed activation in the anterior part of the right visual cortex, but activation was not observed in the injured left visual cortex. Moreover, DTI results revealed the absence of the OR in the left hemisphere [34]. DTI at 12 months after birth revealed the presence of a left hemisphere OR, which became more noticeable at the age of 20 months [16]. The establishment of left hemisphere OR fibers was particularly visible in the anteroposterior direction, suggesting the occurrence of recovery of the injured left OR in the vicinity of the lesion. On 20-month fMRI, the strongest OR activation was observed in the anterior part of the primary visual cortex, and significant OR activation was also observed in the lingual gyrus below the interhemispheric fiber tracts from the forceps major as well as close to the ipsilateral OR fibers. As a result, the authors concluded that the follow-up fMRI and DTI data for this patient indicated functional cortical recovery concurrent with the structural recovery of the injured left OR. However, DTT for the OR was reconstructed by applying a deterministic algorithm using the streamline-like approach (DTT reconstruction program: undescribed, reconstruction conditions: anisotropy threshold of 0.27 and angles less than 40°), and the authors presented the configurational changes of the OR without providing the relevant results for the DTT parameters [28–30]. In addition, their observations were for a single case, and MRI machine and imaging acquisition parameters at three months after birth were different with those at 12 and 20 months after birth.

**Figure 3:**
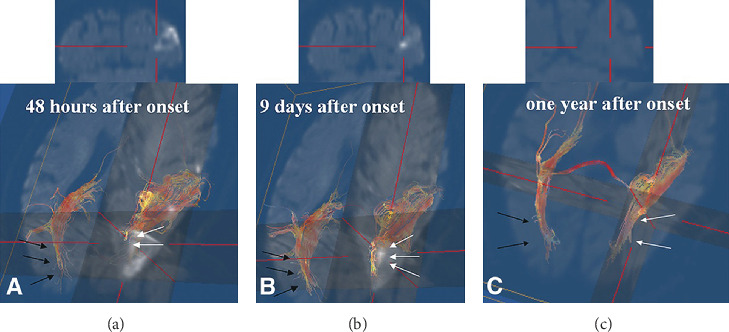
Diffusion tensor imaging (DTI) performed two days after onset (a), nine days after onset (b), and one year after onset (c). (a) The optic radiation reaches the occipital pole in the right hemisphere (black arrow) but is completely interrupted in the left hemisphere, corresponding to a high-intensity area that appears like a white cloud caused by the local edema (white arrow). (b) The left optic radiation is interrupted to a lesser extent at the left cortical lesion (white arrow). (c) There is no longer an interruption visible in the left optic radiation (the optic radiation was reconstructed by using a deterministic algorithm: VOLUME-ONE V1.56 software program (reprinted with permission from Yoshida et al., J *Neuroophthalmol* 2006; 26: 11-17).

In 2006, Yoshida et al. reported on a 68-year-old patient who presented with an injured OR following infarction in the left occipital lobe including the OR [17]. The right homonymous hemianopic paracentral scotomas were markedly improved on visual field test results obtained at one month after onset compared with those at onset. Follow-up fMRI scans, which were acquired four times (two days, nine days, 30 days, and one year after onset), revealed a progressive increase in the activated areas within the lesioned left occipital lobe and a progressive decrease in the activated areas in the nonlesioned right occipital lobe. On follow-up DTTs (DTI: 1.5T clinical scanner (Magnetom Vision, Siemens, Erlangen, Germany) using single-shot SE-EPI sequence, DTT: deterministic algorithm, VOLUME-ONE V1.56 software program, the diffusion TENSOR visualizer (dTV) V1.5 software program, and reconstruction conditions: undescribed), the left OR was completely interrupted at the infarct lesion area on the 2-day postonset DTT and interrupted to a lesser degree on the 9-day postonset DTT. Subsequently, on the 1-year postonset DTT, no interruption of the left OR was observed (Figure 3). Consequently, the authors of this case study had demonstrated recovery of an injured left OR by using follow-up fMRI and DTI/DTT [17]. However, the authors described the configurational changes of the OR on DTI color maps without presenting the DTT parameter values. Furthermore, the study was limited because it was a single case report.

Subsequently, Govindan et al. demonstrated reorganization of injured ORs in ten patients (age range: 4-19 years, mean age: 12.3 ± 4.3 years) that underwent occipital lobectomy [18]. The authors recruited ten pediatric patients who had undergone surgical resection of the unilateral occipital cortex due to intractable epilepsy. Within the surgical group, the FA values of the OR contralateral to the resection side demonstrated a positive correlation with the duration of the period between surgery and DTI (1.5T scanner (General Electric system; General Electric Company, Fairfield, CT), DTT: DTI-Studio software version 2.40, and reconstruction conditions: anisotropy threshold of 0.2 and angle less than 60°) scanning. The results showed that the FA value of the contralateral OR in the surgical group underwent a linear increase during the period from surgical resection of the contralateral occipital lobe to the time of DTI scanning. The authors assumed that the increased FA value in the nonlesioned ORs of the surgical group might have resulted from the formation of additional axonal connections such as by axonal sprouting in the contralateral OR after surgical resection of the unilateral occipital cortex. Consequently, the authors suggested that the contralateral OR underwent significant changes in anisotropy after surgery and that the structural white matter changes might be the result of plasticity changes following the unilateral occipital ablation [18]. However, this study determined the changes in FA values by using the ROI-based method, which could yield false results due to variability in the assigning of ROI location [27].

In 2010, Chen et al. reported on the recovery of injured ORs in sixteen patients (age range: 16-67 years, mean age: 40 years) with visual pathway lesions who received hyperbaric oxygen (HBO) treatment [19]. Sixteen patients with unilateral or bilateral visual disorders caused by visual pathway lesions (two patients, contusion of optic nerve; two patients, optical neuritis; two patients, optical glioma; two patients, contusion of occipital lobe; two patients, wounded hematoma of occipital lobe; four patients, infarction of occipital lobe; and two patients, inflammation of temporal-occipital lobe) underwent HBO treatment (three courses/day at two atmospheres absolute for 20 minutes over a ten-day period). Pre-HBO treatment fMRI revealed fewer activated voxels in the patient group than in the study's control group. By contrast, on post-HBO treatment fMRI, there was an increase in the number of activated voxels in the patient group compared to that before HBO treatment, and the number of voxels after HBO treatment of the patient group was not different from that of the control group. On pre-HBO treatment DTI, the FA values of three ROIs in the ORs of the patient group were lower than those of the control group. In contrast, on post-HBO treatment DTI, the FA values had increased but remained below those of the control group. In addition, decreased numbers or interruptions of OR fibers in the patient group were detected on pre-HBO-treatment DTI (a single-shot SE-EPI sequence) and DTT (deterministic algorithm: dTV. II.R2 software Volume-one 1.72 (the Laboratory of Image Calculation and Analysis, Tokyo University), reconstruction conditions: anisotropy threshold of 0.18). However, the post-HBO treatment DTI or DTT results showed that the number of white matter fiber tracts increased after the HBO treatment, although the fiber changes in some cases were not remarkable. As a result, the authors concluded that the FA increase following HBO treatment in the patient group was suggestive of the occurrence of OR injury recovery, which was indicated by increases in the directionality and integrity of the visual fibers that were paralleled by the patients' cortical function improvements [19]. However, this study included patients with other visual pathway lesions in addition to the OR lesions. Furthermore, the study determined the changes in FA values using the ROI-based method, which can yield false results due to variability in the assigning of the ROI; in addition, DTT of the OR was reconstructed using a deterministic algorithm [27–30].

In 2011, Polonara et al. demonstrated the recovery of an injured OR in a 24-year-old patient with a cerebral infarct [23]. The patient presented with sudden hemianopia and paresthesia due to cerebral infarction in the right thalamus and occipital lobe (the end portion of the OR and primary visual cortex). On follow-up visual field tests, the bilateral left homonymous hemianopsia exhibited partial recovery at one month after onset compared to that during the acute phase. Although the lesioned right primary visual cortex was not shown as activated on acute-phase fMRI performed following peripheral left visual field stimulation, this area was shown as activated on one-month postonset fMRI. For reconstruction of the OR, ROIs were placed in the occipital lobes at the sites of the fMRI activation foci that had been evoked in the primary visual cortex. The observed decrease in the FA value in the right OR on the acute-phase DTT was shown as almost resolved on the one-month DTT (DTI: 1.5T machine (General Electric Signa Excite, GE Medical Systems, Milwaukee, WI, USA) using single-shot SE-EPI; DTT: deterministic algorithm, FuncTool 3.1.22 (GE Medical Systems); and reconstruction conditions: anisotropy threshold of 0.18). Fewer streamlines were noted in the right OR on the acute-phase DTT, whereas symmetrical configuration of the left and right ORs was observed on the one-month postonset DTT. The authors demonstrated the recovery of a cerebral infarct-injured OR, and they assumed the OR recovery was attributed to the resolution of local factors such as edema and not to changes related to brain plasticity. However, this was a single case report and the DTT of the OR was reconstructed using a deterministic algorithm [27–30].

In 2012, Farbota et al. reported on changes of the regional white matter including the OR with change of the visuomotor speed in 12 patients (mean age: 35.00 ± 12.76 years) with traumatic brain injury [24]. DTI was scanned three times (average two months, one year, and four years after onset; DTI: General Electric 3.0T (Waukesha, WI) MRI system using a cardiac-gated, diffusion-weighted sequence). The authors demonstrated that the longitudinal increase in FA values in the white matter was correlated with the improvements on the visuomotor speed task (the sagittal stratum) and cognition control task (the superior longitudinal fasciculus and OR). Because the increased FA values in the white matter indicated improvement of white matter integrity, the authors suggested that the FA increment reflected a plasticity and their results could provide a physiological basis of rehabilitation. However, this study did not confirm the OR injury initially and could not demonstrate direct relation between the improvement of visual function and changes of the OR. Furthermore, the measurement of the FA value in the regions of interest on DTI can be unreliable, especially in the follow-up study [27].

In 2014, Seo and Jang reported on a 56-year-old patient who showed injured ORs after cerebral infarction in the bilateral occipital lobes [20]. The patient presented with severe visual impairment since infarct onset and the patient noted that she could not see anything, although the central part of the visual field remained, but only dimly, at one week after onset when the patient started rehabilitation. The patient's visual impairment improved with time; at five weeks after onset, the patient reported that her visual field and visual acuity had improved, although her vision was still cloudy. Consequently, she could see a watch at a distance of three meters and could walk independently on an even floor at five weeks after onset. The FA and ADC values for the bilateral ORs and the FN value for the left OR on DTT (DTI: 1.5T Philips Gyroscan Intera (Hoffman-La Roche, Best, Netherlands) with single-shot EPI, navigator echo, DTT: deterministic algorithm, DTI-Studio software (CMRM, Johns Hopkins Medical Institute, USA), and reconstruction conditions: anisotropy threshold of 0.15 and angle less than 70°) one week after onset were similar to those observed on DTT at four weeks after onset; however, the FV of the left OR had increased from 257 (one week postonset) to 353 (four weeks postonset). Grossly, fibers of the left OR also showed an increase on the four-week DTT compared to that on the one-week postonset DTT. On one-week postonset fMRI, no activation was observed in the occipital lobe, including in the primary visual cortex. By contrast, activation of the visual cortex, including the bilateral primary visual cortices, was observed on four-week postonset fMRI. As a result, the authors concluded that they had been able to demonstrate visual recovery in this patient by assessing changes in follow-up DTT and fMRI results; furthermore, the recovery of the left OR appeared to be attributed more to a resolution of local factors, such as changes in peri-infarct edema, than to brain plasticity [20]. However, their study was limited because it was a single case report and the authors employed a deterministic algorithm for reconstruction of the OR [27–30].

In 2015, Jang and Lee, by using follow-up DTTs (DTI: 1.5T Philips Gyroscan Intera (Hoffman-La Roche, Best, the Netherlands) with single-shot EPI, navigator echo, and reconstruction conditions: anisotropy threshold of 0.2) of the OR, reported on a 57-year-old patient with a cerebral infarct in the right temporal lobe who showed recovery of the associated visual field defect via the transcallosal fibers of the corpus callosum [21]. Left-side homonymous hemianopsia was detected on a 2-week postonset visual field test; however, recovery of the left lower peripheral visual defect along with recovery of the upper and medial portions of the initial left hemianopia was observed on the 11-month postonset visual field test results. Both the 2-week and 11-month DTTs for the right OR showed tract discontinuation between the right lateral geniculate nucleus and the right OR. Interestingly, the right posterior OR was observed to be connected to the left OR via the transcallosal fibers, a connection that passed through the splenium of the corpus callosum. In addition, on 2-week postonset DTT, the left OR was shown to be connected to the transcallosal fibers, and on 11-month DTT, these transcallosal fibers appeared to have elongated to the right primary visual cortex via the right posterior OR. The authors suggested that the visual field defect in this patient had recovered via the recovery of the OR injury by way of the transcallosal fibers of the splenium of the corpus callosum [21, 35]. However, this was a single case report and DTT for the OR was reconstructed using a deterministic algorithm [27–30].

Recently, Jang and Seo reported on a 19-year-old patient who recovered from an OR injury that occurred following a hypoxic-ischemic brain injury (HI-BI) induced by hanging [22]. The patient underwent conservative management for HI-BI at a general hospital. At four months after onset, she was transferred to the rehabilitation department of our university hospital. Brain MRI showed leukomalactic lesions in the parieto-temporo-occipital lobes in both hemispheres. The patient was in a vegetative state with a Coma Recovery Scale-Revised (CRS-R) score of 9 (full score: 23) [36]. Although she could open her eyes spontaneously, she did not show a blinking reflex upon visual stimulation. In addition, she did not exhibit visual tracking of visual stimuli and could not identify anything, including members of her family. She underwent comprehensive rehabilitation, which included medication (amantadine, methylphenidate, and baclofen treatments), physical therapy, occupational therapy, transcranial direct current stimulation of the upper occipital area, and repetitive transcranial magnetic stimulation of the right upper occipital area. After one month of rehabilitation, the patient had recovered to a minimally conscious state (CRS-R score: 15). In addition, she showed a blink reflex to visual stimulation and was able to visually track visual stimuli. On 4-month DTT (DTI: 1.5T Philips Gyroscan Intera, reconstruction conditions: anisotropy threshold of 0.15 and angle less than 70°), discontinuations of the OR, with the exception of tiny fibers in the posterior portion of the OR, were observed in both hemispheres. On a 5-month postonset DTT, the OR was shown to be connected to the upper occipital lobe on both sides. As a result, the authors concluded that, following rehabilitation and concurrent with the recovery of the patient's consciousness and visual function, her OR also showed evidence of recovery [22]. However, this was a single case study that used a deterministic algorithm for reconstruction of the OR [27–30].

## 4. Conclusions

In this minireview article, nine DTI-based studies that demonstrated recovery of injured ORs were reviewed [16–24]. The information provided in the reviewed studies suggests that an OR injury has a potential for recovery after injury. The results of these studies can form a basis for the further elucidation of OR injury recovery mechanisms. Based on the results presented in the nine reviewed studies, two recovery mechanisms have been presented: recovery via the original OR pathway and recovery via the transcallosal fibers of the corpus callosum. In addition, these studies indicate that follow-up DTI/DTTs are useful for the demonstration of recovery of an OR injury. However, all DTT studies used a deterministic algorithm which is known to be less accurate than a probabilistic algorithm for reconstruction of Meyer's loop [28–30]. In addition, only nine studies on this topic have been conducted to date and six of those nine studies were case reports. Half of the studies showed results that only reflected configurational changes and did not include data for various DTI parameters. In addition, majority of the reviewed studies used low magnetic field MRI (1.5 T). Because field strength does affect the signal-to-noise ratio (SNR) and artifacts of diffusion-weighted images, the employment of low magnetic field MRI could influence the quantitative and spatial accuracy of DTI [37]. Therefore, further studies involving larger numbers of subjects and using higher magnetic field MRI with probabilistic algorithm are warranted.

## Figures and Tables

**Table 1:** Previous diffusion tensor imaging studies on optic radiation injury recovery.

Authors	Publication year	Number of patients	Duration to DTI	Pathology	DTT analysis method & results	Combined assessment
Seghier et al., (2005)	2005	1	12 & 20 months	Cerebral infarct	Color map (configuration↑)	fMRI
Yoshida et al., (2006)	2006	1	2 & 9 days and 1 year	Cerebral infarct	DTT Deterministic algorithm (configuration↑)	fMRI
Govindan et al., (2007)	2008	10	Pre- & postsurgery	Occipital lobectomy	ROI on DTI (FA↑)	
Chen et al., (2010)	2010	10	Before & after hyperbaric oxygen treatment	Contusion:2 Wounded hemorrhage: 2 Infarction: 4 Inflammation: 2	ROI on DTI (FA↑) DTI and DTT Deterministic algorithm (configuration↑)	fMRI
Polonara et al., (2011)	2011	1	Acute phase & 1 month	Cerebral infarct	DTT Deterministic algorithm (FA↑, configuration↑)	fMRI
Farbota et al., (2012)	2012	12	2 months, 1 & 4 years	Traumatic brain injury	DTI (FA↑: superior longitudinal fasciculus and optic radiation)	Neuropsychological test
Seo and Jang, (2014)	2014	1	1 & 4 weeks		DTT Deterministic algorithm (FA-, ADC-, FV↑)	fMRI
Jang and Lee, (2015)	2015	1	2 weeks & 11months	Cerebral infarct	DTT Deterministic algorithm (transcallosal fibers: configuration↑)	
Jang and Seo, (2019)	2019	1	4 & 5 months	Hypoxic-ischemic brain injury	DTT Deterministic algorithm (configuration↑)	

DTI: diffusion tensor imaging; DTT: diffusion tensor tractography; fMRI: functional magnetic resonance imaging; ROI: region of interest; FA: fractional anisotropy; ADC: apparent diffusion coefficient; FV: fiber voxels. Arrow directions: upward indicates an increase; downward indicates a decrease.
